# Socioeconomic inequity in coverage and quality of maternal postnatal care in Ethiopia

**DOI:** 10.1111/tmi.13829

**Published:** 2022-12-14

**Authors:** Emma Beaumont, Della Berhanu, Elizabeth Allen, Joanna Schellenberg, Bilal Iqbal Avan

**Affiliations:** ^1^ Department of Infectious Disease Epidemiology Faculty of Epidemiology and Population Health, London School of Hygiene and Tropical Medicine London UK; ^2^ Department of Disease Control London School of Hygiene and Tropical Medicine London UK; ^3^ Health System and Reproductive Health Research Directorate, Ethiopian Public Health Institute Addis Ababa Ethiopia; ^4^ Department of Medical Statistics, Faculty of Epidemiology and Population Health London School of Hygiene and Tropical Medicine London UK; ^5^ Department of Population Health Faculty of Epidemiology and Population Health, London Schoold of Hygiene and Tropical Medicine London UK

**Keywords:** inequity, maternal health, postnatal care, quality of care

## Abstract

**Objective:**

High‐quality postnatal care is vital for improving maternal health. This study examined the relationship between household socioeconomic status and both coverage and quality of postnatal care in Ethiopia.

**Method:**

Cross‐sectional household survey data were collected in October–November 2013 from 12 zones in 4 regions of Ethiopia. Women reporting a live birth in the 3–24 months prior to the survey were interviewed about the care they received before, during and after delivery and their demographic characteristics. Using mixed effect logistic and linear regression, the associations between household socioeconomic status and receiving postnatal care, location of postnatal care (health facility vs. non‐health facility), cadre of person providing care and the number of seven key services (including physical checks and advice) provided at a postnatal visit, were estimated.

**Results:**

A total of 16% (358/2189) of women interviewed reported receiving at least one postnatal care visit within 6 weeks of delivery. Receiving a postnatal care visit was strongly associated with socioeconomic status with women from the highest socioeconomic group having twice the odds of receiving postnatal care compared to women in the poorest quintile (OR [95% CI]: 1.98 [1.29, 3.05]). For each increasing socioeconomic status quintile there was a mean increase of 0.24 postnatal care services provided (95% CI: 0.06–0.43, *p* = 0.009) among women who did not give birth in a facility. There was no evidence that number of postnatal care services was associated with socioeconomic status for women who gave birth in a facility. There was no evidence that socioeconomic status was associated with the provider or location of postnatal care visits.

**Conclusion:**

Postnatal care in Ethiopia shows evidence of socio‐economic inequity in both coverage and quality. This demonstrates the need to focus on quality improvement as well as coverage, particularly among the poorest women who did not deliver in a facility.

## INTRODUCTION

Good postnatal care (PNC) is vital for improving maternal health. An estimated 60% of maternal deaths in high, middle and low‐income countries occur during the postnatal period, defined as the 6 weeks after delivery [1, 2]. Efforts to improve global maternal health have been given priority, featuring in both the Millennium Development Goals and the more recent Sustainable Development Goals [3, 4]. Although this led to major improvements in global maternal mortality and morbidity, the goals have not been met, particularly in sub‐Saharan Africa and among certain disadvantaged populations [2, 5]. This is in part due to the stagnation of improvements in PNC [6]. Coverage of PNC continues to be among the lowest of all maternal health indicators [7, 8], particularly in sub‐Saharan Africa [2, 6] and among women living in rural areas with lower socioeconomic status (SES) and education [5].

While data on the number of postnatal contacts are important, they do not describe whether maternal healthcare needs are being met by these contacts. High quality of care during PNC visits is also required to reduce the burden of mortality and morbidity [9]. There is a lack of discussion around quality of maternal PNC services [10] and of studies focusing on this.

More recently there has been a push to define what quality of maternal care means, with WHO releasing *Standards for Improving Quality of Maternal and Newborn Care in Health Facilities* in 2016 [11]. They recommend that all mothers receive at least four PNC visits at the following time points: within 24 h, 48–72 h, 7–14 days and 6 weeks of delivery. The report includes recommendations for the content of maternal PNC visits, which include assessments of both physical and mental health as well as counselling on health‐related practices such as identifying danger signs, nutrition and hygiene [12].

In low‐ and middle‐income settings high household SES [5, 13, 14, 15, 16] is associated with receiving PNC. Other demographic and reproductive characteristics have also been reported to be associated with receiving PNC [5, 13, 14, 15, 16, 17, 18, 19, 20]. However, we found no studies that look at these factors in relation to the quality of PNC.

Ethiopia is one of the most populous countries in sub‐Saharan Africa, with a population estimated at over 100 million people in 2020 [21, 22], and is also one of the poorest and least urbanised [23]. The country continues to be a major contributor to worldwide maternal deaths [24]. In 2016, the maternal mortality ratio was 412 deaths per 100,000 live births [23], which is higher than the global average but an improvement from 673 deaths per 100,000 live births in 2005 [25]. The Ethiopian Demographic and Health Survey reported an increase of women receiving PNC within 6 weeks of delivery from 6% in 2005 to 20% in 2016. In recent years, the Ethiopian government has increased its focus on improving maternal and newborn outcomes through strengthening of its health systems. However, whether these improvements are reaching the poorest in the community to the same degree as the least poor still needs to be explored. Using cross‐sectional data from a large household survey conducted in four regions of Ethiopia, this study aimed (a) to understand whether coverage and quality of maternal PNC in Ethiopia differ by household SES and (b) to identify the health system and maternal demographic and reproductive factors that influence this relationship.

## METHODS

### Study design and setting

We used cross‐sectional data collected as part of the baseline survey of the quasi‐experimental evaluation study of the Community Based Newborn Care programme conducted in October–November 2013. The household survey was conducted in 12 zones across 4 regions in Ethiopia (Amhara: *n* = 2 zones, Oromia: *n* = 4, SNNP: *n* = 4 and Tigray: *n* = 2) [26].

In Ethiopia, zones are divided up into districts which are further divided into *kebeles* (villages) and *gotes* (sub‐village). At the time of the survey, primary health services were provided through the district level Primary Health Care Units (PHCU). Each district contained five PHCUs and one hospital, which served approximately 100,000 people [27, 28]. A single PHCU was comprised of five health posts and a referral health centre. Each health post was staffed by two community health workers known as health extension workers; health centres were staffed by health officers and nurses; and the referral hospital was staffed by doctors, health officers and nurses.

### Sampling and participants

The sampling procedure for this study [29] used a multi‐stage sampling approach. A total of 101 districts were selected from the 12 zones and then 209 PHCUs were selected from these districts. Within each PHCU, one *kebele*, and within this *kebele* one *gote* were selected using simple random sampling. Within the selected *gote*, 50 households were randomly selected and all consenting female household members of reproductive age (15–49 years) were interviewed about their reproductive history. All women who reported having a live delivery within 3–24 months before the interview were included in the analysis. If women reported two live deliveries in the period, the data from the most recent delivery were retained for analysis.

### Data collection

Women were asked about their health‐seeking behaviours and the care they received, comprising details of the timing, location, provider and content of up to three PNC visits. Data on demographic characteristics and household socio‐economic status were also collected. Data were obtained by trained data collectors using paper forms which were then double‐entered into an electronic database using CSPro (version 7.1.1, United States Census Bureau, https://www.census.gov/data/software/cspro.html).

### Data management

For socio‐demographic variables an indicator of relative household SES was constructed using principal components analysis applied to proxy variables for household wealth. These were: material used for the roof, water supply, type of toilet, electricity and ownership of a radio, mobile phone, bed, lamp and watch. Maternal education was excluded from the indicator as we wished to explore its effects on the outcome separately. The resulting continuous score was divided into quintiles of households from quintile 1 (most poor) to quintile 5 (least poor).

For PNC variables, maternal PNC was defined as a health check performed by either clinical staff (doctor, health officer, nurse) or non‐clinical health extension worker within 6 weeks after delivery, as reported by the mother.

Quality of PNC was assessed through the following dimensions: timing of the first visit (within 7 days of delivery vs. >7 days post‐delivery), location of first PNC visit (facility [hospital or health centre] versus non‐facility [health post or woman's household]), provider of first PNC (clinical vs. non‐clinical staff) and visit content. The quality of PNC content was defined by the number of seven key services provided during a visit as reported by the mother: checking breasts, blood pressure and delivery‐related wounds; advice on breast feeding, maternal danger signs, family planning and maternal nutrition. These services were part of the training for HEWs and are also included in the health centre staff PNC training. Although the PNC training is very similar for HEWs and clinical staff, access to certain equipment may differ. Data on location and provider of PNC were taken from the first visit only.

### Statistical analysis

Demographic and reproductive characteristics were summarised for all eligible women. Continuous variables were summarised using means and standard deviations. Categorical variables were summarised using numbers and percentages.

For the binary outcomes, occurrence of PNC, receiving PNC within 7 days, first PNC visit at a health care facility and first PNC visit provided by clinical staff, the percentage and 95% confidence interval (95% CI) were calculated stratified by SES quintile and delivery location (facility vs. non‐facility). For the continuous outcome, number of services provided, the mean and 95% CI stratified by SES quintile and delivery location are presented.

Descriptive statistics were calculated for all outcomes, number and percentages for binary outcomes and mean and standard errors (SE) for continuous outcomes. These were plotted graphically, stratified by delivery location. Associations between SES quintile and the occurrence, location, provider and content quality of visits were calculated using univariate and multivariate mixed‐effects models with a random effect for PHCU. For binary outcomes mixed‐effect logistic models were used and the odds ratios (OR) and 95% CI were calculated. The number of services provided during a visit was treated as a continuous outcome and was measured at each individual PNC visit. Therefore, mixed‐effect linear regression models included random effects for PHCU and the woman. The mean difference and 95% CI were calculated. None of the women in the analysed population were from the same household, so we did not adjust for clustering by household in this analysis.

The association between SES and receiving each of the seven key PNC services independently was calculated using a mixed‐effect logistic model treating SES as a continuous variable and stratified by delivery location.

Likelihood ratio tests were used to test for effect modification by delivery location of the association between SES and all outcomes. Demographic and health system characteristics were considered as potential confounders and adjusted for if they were associated with both SES and quality of PNC content and not considered to be on the causal pathway.

Linearity of the association of SES quintiles with each outcome was assessed using likelihood ratio tests comparing a model which treated SES quintiles as a continuous variable with a model treating SES quintiles as categorical. All analysis was completed using Stata version 15.

## RESULTS

Residents of 10,224 households were asked about their household's sociodemographic characteristics. All women (10,999) of reproductive age (15–49 years) in these households were interviewed about their reproductive history and of these women 20% (2189) reported a live delivery in the 3–24 months before the interview. Twenty‐two women reported a non‐live birth outcome and were excluded from further analysis.

Women who had a live birth 3–15 months prior to this survey had a mean age of 28 (standard deviation [SD] 6) years and 2 (SD 3) years of formal education. Eighty‐eight percent were married, 77% were Christian and 17% were Muslim. Seventy‐nine percent had given birth previously (Table 1).

**TABLE 1:** Population characteristics

Characteristic	Women with full term delivery, *N* = 2189	Received PNC, *N* = 358
*Demographic*		
Maternal age (years), mean (SD)	27.8 (6.3)	27.4 (6.2)
Missing*, n* (%)	120 (6)	14 (4)
Maternal education (years), mean (SD)	2.1 (3.2)	2.8 (3.4)
Missing*, n* (%)	117 (5)	13 (4)
Marital status, *n* (%)		
Currently married	1917 (88)	319 (89)
Not currently married	160 (7)	24 (7)
Missing	112 (5)	15 (4)
Religion, *n* (%)		
Christian	1684 (77)	276 (77)
Muslim	373 (17)	67 (19)
Other	21 (1)	2 (1)
Missing	111 (5)	13 (4)
Household socioeconomic status (SES) quintile, *n* (%)		
Most poor: 1	502 (23)	62 (17)
2	375 (17)	40 (11)
3	461 (21)	75 (21)
4	433 (20)	80 (22)
Least poor: 5	418 (19)	101 (28)
*Reproductive*		
Parity, *n* (%)		
1 birth	457 (21)	86 (24)
>1 birth	1726 (79)	272 (76)
Missing	6 (0.3)	0 (0)
*Health system*		
Delivery location, *n* (%)		
Facility	447 (20)	135 (38)
Non facility	1730 (79)	222 (62)
Missing	12 (1)	1 (0.3)
Delivery by caesarean, *n* (%)		
Yes	47 (2)	20 (6)
No	2117 (97)	334 (93)
Missing	25 (1)	4 (1)
1st PNC visit location, *n* (%)		
Home	‐	116 (32)
Health post	‐	89 (25)
Health centre	‐	121 (34)
Hospital	‐	18 (5)
Missing	‐	14 (4)
1st PNC visit provider, *n* (%)		
HEW	‐	203 (57)
Nurse	‐	134 (37)
Health officer/Doctor	‐	21 (6)
Number of PNC visits, *n* (%)		
1	‐	193 (54)
2	‐	93 (26)
>2	‐	72 (20)
1st PNC visit timing (days after delivery), mean (SD)	‐	12.7 (13.9)
Timing of first PNC visit, *n* (%)		
≤1 day post‐delivery	‐	99 (28)
2–7 days post‐delivery	‐	103 (29)
8–42 days post‐delivery	‐	156 (44)

At least one PNC visit within 6 weeks post‐delivery was reported by 16% (358). Of these women, 28% (99) received PNC within 24 h of delivery, 29% (103) within 2–7 days and 44% (156) within 8–42 days. The proportion of women receiving PNC within the different the demographic, parity and health system categories presented in Table 1 were similar, ranging from 16% to 19% with the exception of facility delivery. Thirty percent of the women who delivered in a facility received maternal PNC, whereas only 13% of women did not deliver in a facility did.

**FIGURE 1:**
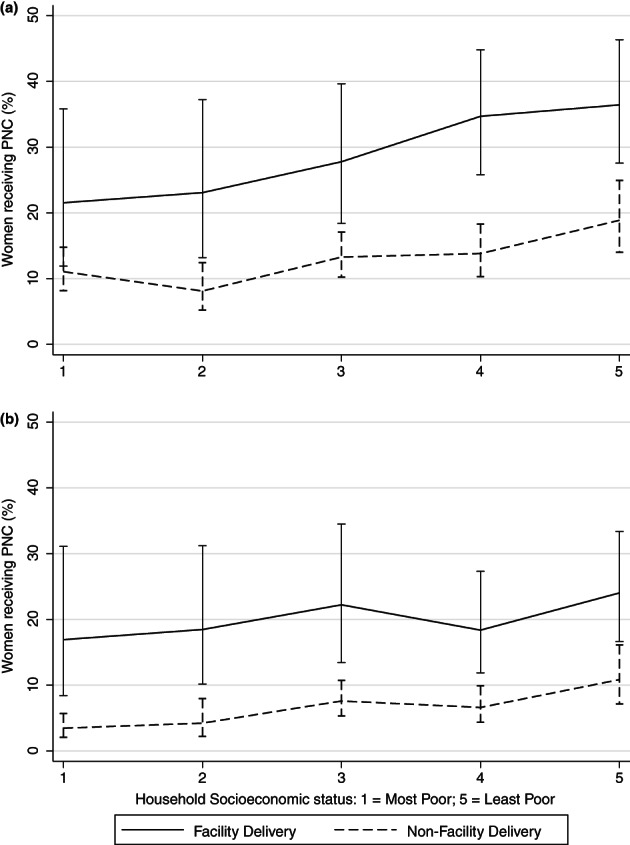
Percentage and 95% CI of women receiving maternal postnatal care (PNC) within (a) 6 weeks, and (b) 7 days of delivery by household socioeconomic status quintile and location of delivery. (a) OR (95% CI) comparing least poor with most poor SES quintile: 1.98 (1.29, 3.05), *p* = 0.0008, *N* = 2177, adjusted for delivery location. Likelihood ratio test for interaction by delivery location *p* = 0.70. (b) OR (95% CI) comparing least poor with most poor SES quintile: 2.71 (1.53, 4.80), *p* = 0.004, *N* = 2177, adjusted for delivery location. Likelihood ratio test for interaction by delivery location *p* = 0.67.

The proportion of women receiving at least one PNC visit increased with increasing SES. Among women who did not deliver in a facility, 11% in the poorest quintile received PNC vs. 19% in the least poor quintile. Among women who did deliver at a facility, 22% of women in the poorest quintile received PNC vs. 36% in the least poor quintile (Figure 1a). Delivery location was strongly associated (*p* < 0.0001) with receiving both PNC and SES but there was no evidence that delivery location modified the effect of SES on receiving PNC (*p* = 0.70). After adjusting for delivery location, women from the highest socioeconomic group had twice the odds of receiving PNC as women in the poorest quintile (OR [95% CI]: 1.98 [1.29, 3.05]).

The mean (SD) number of days after delivery at which the first PNC visit occurred was 12.7 (13.9) and 56% of women who received PNC had a visit within 7 days of delivery (Table 1). Receiving PNC within 7 days of delivery was associated with both higher SES and delivering in a facility. There was no evidence that delivery location modified the effect of SES on receiving PNC within 7 days of delivery (*p* = 0.67). Women in the least poor SES quintile had more than twice the odds of receiving a PNC visit within 7 days of delivery as the most poor after adjusting for delivery location (OR [95% CI]: 2.71 [1.53–4.80]; Figure 1b). However, there was no evidence of a linear trend in the association between increasing SES and PNC within 7 days of delivery.

Over half (54%) of the 358 women who received PNC reported only receiving one visit in the 6 weeks' postnatal period and 20% received three or more visits (Table 1). A health extension worker provided 57% of first PNC visits, nurses provided 37%, and a doctor or health officer provided 6%.

Fifty‐seven percent of women received PNC at home or at a health post (Table 1). Ninety‐three percent of women received all their reported PNC in the same type of location and 94% by a healthcare worker of the same cadre. There was no evidence that the number of women who received PNC in different locations or by different providers differed by SES.

Delivering in a facility was strongly associated with receiving PNC from clinical staff (OR [95% CI]: 7.42 [3.74–14.72], *p* < 0.001). Delivering in a facility was also strongly associated with receiving PNC in a health facility (OR [95% CI]: 11.80 [5.57–25.00], *p* < 0.001). A higher proportion of women who received their first PNC visit at a facility were first‐time mothers (29%) than of women who received their first PNC visit elsewhere (20%) (Table S1). There was no evidence that provider or location of PNC was associated with SES (Table S2), or services provided during a PNC visit (data not shown).

**FIGURE 2:**
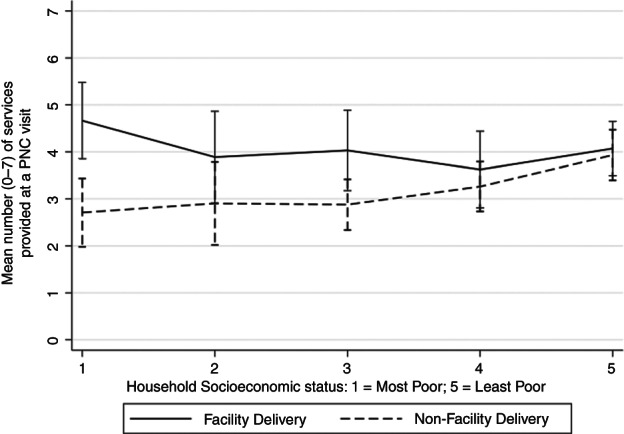
Mean number (0–7) and 95% CI of services provided at a postnatal care (PNC) visit, by household socioeconomic status (SES) quintile and delivery location. *Facility delivery*: Mean difference (95% CI): −0.12 (95% CI−0.37, 0.14), *p* = 0.37. *Non‐facility delivery*: Mean difference (95% CI): 0.24 (0.06, 0.43), *p* = 0.009. The mean difference presented represents the relative increase or decrease in the mean number of services provided for each increasing SES quintile. Likelihood ratio test for interaction by delivery location: *p* = 0.02.

The 358 women who received PNC had a total of 483 PNC visits. There was evidence of a linear association between SES and the number of services provided and evidence (*p* = 0.02) that this association was modified by location of delivery (Figure 2). Among women in the most poor SES quintile, women who delivered in a facility received a mean of 4.7 services at a PNC visit, whereas women who did not deliver in a facility received a mean of 2.7 services. Women in the least poor SES quintile received a mean of 4.1 services at a PNC visit when they delivered in a facility and 3.9 when they did not deliver in a facility. For each increasing SES quintile the mean difference in number of services increased by 0.24 (95% CI: 0.06, 0.43, *p* = 0.009) among women who did not give birth in a facility. There was no evidence that the number of services provided at a PNC visit was associated with SES for women who gave birth in a facility (mean difference: −0.12 (95% CI: −0.37, 0.14), *p* = 0.37; Figure 2). This trend was also seen when each service was analysed individually. Among women who delivered in a facility, there was evidence that higher SES was associated with the following being included in the PNC visit: checking of breasts and advice given on family planning, maternal nutrition and maternal danger signs. There was no evidence that any of the individual PNC services were associated with SES among women who delivered in a facility (Table 2).

**TABLE 2:** Association between household socioeconomic status (SES) quintile and provision of the seven key services provided at a postnatal care visit, by delivery location

	Facility delivery			Non‐facility delivery		
PNC content	*N* = 186 *n* (%)	OR (95% CI)	*p*‐value	*N* = 296 *n* (%)	OR (95% CI)	*p*‐value
Check breasts	136 (73)	0.70 (0.36–1.35)	0.29	172 (58)	1.58 (1.00–2.49)	0.05
Advise on breast feeding	139 (75)	0.59 (0.29–1.23)	0.16	196 (66)	1.33 (0.83–2.11)	0.23
Advised on maternal danger signs	84 (45)	0.90 (0.38–2.15)	0.82	105 (35)	1.98 (0.98–4.00)	0.06
Family planning	112 (60)	0.68 (0.32–1.45)	0.32	176 (59)	1.91 (1.07–3.71)	0.03
Advised on maternal nutrition	129 (69)	0.65 (0.31–1.35)	0.25	169 (57)	2.25 (1.27–3.99)	0.005
Measured blood pressure	100 (54)	1.17 (0.54–2.56)	0.69	88 (30)	1.48 (0.79–2.76)	0.22
Checked and treated delivery related wounds	42 (23)	Small numbers		37 (13)	Small numbers	

*Note*: The odds ratio (OR) presented represents the relative increase or decrease in odds of receiving the service for each increasing SES quintile.

There was no evidence of an association between the health system, maternal demographic or reproductive characteristics in Table 1 and number of services provided at a PNC visit. Higher maternal education was strongly associated with both higher SES and delivering in a facility; however, adjusting for maternal education did not alter the mean difference in number of services between SES quintiles (data not shown).

## DISCUSSION

WHO defines quality of care as being safe, effective, timely, efficient, equitable and people‐centred [30]. In this analysis, we focused on the socioeconomic inequity of occurrence and timing of maternal PNC contacts, skill level of the provider, location and services provided.

Coverage of PNC in this population was universally low with only 16% of mothers receiving at least one visit within the 6 week postnatal period. Both receiving PNC and receiving a higher number of services during a PNC visit were strongly associated with higher SES. While the proportion of women receiving PNC was higher among women who gave birth in a hospital or health centre, facility delivery did not modify the association with SES. However, the association between number of services provided and SES was modified by location of delivery, with socioeconomic inequity in the number of services provided during PNC only observed among women who did not deliver in a health facility. We found no evidence of an association between SES and the number of PNC visits, location or provider of PNC.

While improving equity in quality of PNC is key to reducing maternal mortality, the low coverage of maternal PNC in the population must be addressed. In this study, 16% of women received at least one PNC visit and there is a strong association between higher SES and receiving PNC. The low coverage of PNC is in line with the Ethiopian Demographic and Health Survey, which reported an increase from 6%, in 2005, to 20%, in 2016, of women receiving PNC within 6 weeks of delivery. However, this includes care given by traditional birth attendants [23, 25] whereas it is access to skilled care which improves maternal health outcomes [2, 9]. Coverage of PNC was higher among women who delivered in a health facility, those who lived in urban areas and were of a high SES. Women of a lower SES also experienced higher levels of discrimination and disrespect at facilities, which affected their quality of care and their likelihood to access it [5, 10, 31]. Indeed the Ministry of Health in Ethiopia has specifically highlighted a need for improvements in quality and equity of health care as well as a move towards more respectful compassionate care in their 2015 Health Sector Transformation Plan [32]. In this analysis, women who received PNC were also more likely to have delivered in a facility and to have more years of education. These findings are consistent with other similar surveys conducted in Ethiopia [17, 23, 25].

Although both coverage and the provider of maternal and newborn PNC are well‐reported in the Ethiopian Demographic and Health Survey, the services provided during a visit are only reported for newborn PNC. The current study highlights that provision of these seven key services during maternal PNC (checking breasts, blood pressure and delivery‐related wounds, advise on breastfeeding, maternal danger signs, family planning and maternal nutrition) are not universally provided even among the least poor. Measuring blood pressure and advising on danger signs is alarmingly uncommon with less than 40% of the study population receiving these services. The two leading causes of maternal mortality in Ethiopia, postpartum haemorrhage and hypertensive disorders, account for almost half of maternal deaths [33, 34]. The low coverage of these essential services could provide a partial explanation for the continuing high maternal mortality rates in the country.

Timing of PNC is also vital for preventing maternal death. In Ethiopia, an estimated 65% of maternal deaths occur within 7 days of delivery, with 45% occurring within the first 24 h [35]. Forty‐three percent of women in this study did not receive a PNC visit within 1 week of delivery and therefore missed this crucial period when the majority of deaths could be prevented. Furthermore, the least poor women had twice the odds of an early PNC visit as the most poor, demonstrating socioeconomic inequity in timely maternal PNC. Higher SES, education and facility delivery have all been previously associated with receiving PNC [5, 13, 14, 15, 16, 17, 18, 19, 20] in low‐ and middle‐income settings. However, we found no studies, which focus on the quality of PNC. Researchers have also reported associations between maternal age, marital status, urban living, parity and antenatal care (ANC) [5, 13, 14, 15, 16, 17, 18, 20] with receiving PNC. We did not assess urban vs. rural living as all women were from a rural setting. We did not find any evidence of an association between maternal age, marital status or parity with quality of PNC, but 90% of the women included in this analysis were currently married, making it unlikely that we would identify any smaller effects should they exist.

When addressing the quality of maternal care, few studies report on PNC, tending to focus on ANC instead. ANC quality was reported to lag behind coverage, particularly in low‐income settings and among those with low SES [36, 37]. High‐quality ANC is associated with facility delivery and the presence of a skilled birth attendant [18, 38, 39, 40] and adequate ANC has a positive impact on birth outcomes, use of paediatric care and immunisations [41, 42, 43]. These findings highlight the importance of measuring quality of visit content as well as coverage when evaluating care. This measurement needs to be extended to PNC.

There are several limitations to this study. The sample size of 358 women reporting post‐natal care is relatively small, making it unlikely that smaller effect sizes would be detected. It was only possible to collect data from women who survived the postnatal period, women who died are most likely to have been from a lower SES [5] and therefore their exclusion from the analysis could have reduced our estimation of the effect size. Births from different parities were grouped in this analysis, and although we found no association between parity and quality of PNC in this dataset an association could have been detected with a larger sample size. Although there is evidence to suggest women are able to accurately report on indicators of PNC [44], respondents' recall could also be associated with maternal education and newborn or child death leading to some bias. Evidence on the effect that recall period has on indicator accuracy is varied; some authors report that consistency and particularly specificity decreases even after 6 weeks [45], other authors suggest a recall period of 2 years is short enough to not hamper accurate recall [46]. Caution should also be used when generalising these results to all of Ethiopia, as zones included in the analysis were not randomly selected and therefore not a representative sample of the regions.

Further work is needed to understand mechanisms behind inequity of maternal PNC quality, particularly among women who do not deliver at a facility. Understanding the role of continuum of care from ANC to PNC to ensuring equitable quality of care would also be useful. This article focuses solely on maternal PNC. However, coverage of newborn PNC is also very low, particularly in sub‐Saharan Africa. More work is needed to understand the role of socioeconomic inequity in both coverage and quality of newborn PNC and how this relates to maternal PNC.

## CONCLUSION

This article presents an analysis of the socioeconomic inequity of maternal PNC quality in Ethiopia and highlights the need to focus on quality improvement, particularly among women of lower SES who do not deliver in a facility. Further research in this area may help explain why maternal mortality and morbidity remains high in low‐income settings and among certain disadvantaged populations.

## FUNDING INFORMATION

This project was funded by Bill & Melinda Gates Foundation (https://www.gatesfoundation.org/; INV‐007644). The funder had no role in the study design, data collection and analysis, decision to publish, or preparation of the manuscript.

## Supporting information


**Data S1** Supporting Information

## Data Availability

Fully anonymised study datasets and survey questionnaires are available upon request on the London School of Hygiene and Tropical Medicine's data repository, Data Compass (https://datacompass.lshtm.ac.uk/id/eprint/1112/).
