# Supplementary material for: Socioeconomic inequity in coverage and quality of maternal postnatal care in Ethiopia
Source: Trop Med Int Health. 2022 Dec 14;28(1):25–34. doi: 10.1111/tmi.13829 (PMC10108216; doi:10.1111/tmi.13829)
Supplement: Supplementary file 1 — Data S1 Supporting Information [file TMI-28-25-s001.docx]

**Supplementary Tables and Figures**

**Supplementary Table 1** Baseline Characteristics by location of first PNC visit

| **Characteristic** | **Received first PNC visit in a non-facility**  **N=203** | **Received first PNC visit in a facility**  **N=141** |
| --- | --- | --- |
| **Demographic** | | |
| Maternal age (years), mean (sd) | 27.8 (6.1) | 27.2 (6.3) |
| *Missing, n(%)* | 6 (3) | 8 (6) |
| Maternal education (years), mean (sd) | 2.6 (3.3) | 3.0 (3.7) |
| *Missing, n(%)* | 5 (2) | 8 (6) |
| Marital status, n(%) |  |  |
| *Currently Married* | 184 (91) | 123 (87) |
| *Not Currently Married* | 14 (7) | 9 (6) |
| *Missing* | 5 (2) | 9 (6) |
| Religion, n(%) |  |  |
| *Christian* | 168 (83) | 96 (68) |
| *Muslim* | 30 (15) | 35 (25) |
| *Other* | 0 (0) | 2 (1) |
| *Missing* | 5 (2) | 8 (6) |
| Household socioeconomic status quintile, n (%) |  |  |
| *Most poor: 1* | 43 (21) | 19 (13) |
| *2* | 18 (9) | 20 (14) |
| *3* | 44 (22) | 28 (20) |
| *4* | 48 (24) | 28 (20) |
| *Least poor: 5* | 50 (25) | 46 (33) |
| **Reproductive** | | |
| Parity, n(%) |  |  |
| *1 birth* | 40 (20) | 41 (29) |
| *>1 birth* | 163 (80) | 100 (71) |
| *Missing* | 0 (0) | 0 (0) |
| **Health System** | | |
| Delivery location, n(%) |  |  |
| *Facility* | 41 (20) | 91 (65) |
| *Non Facility* | 161 (79) | 50 (35) |
| *Missing* | 1 (0.5) | 0 (0) |
| Delivery by caesarean, n(%) |  |  |
| *Yes* | 1 (0.5) | 17 (12) |
| *No* | 202 (99.5) | 120 (85) |
| *Missing* | 0 (0) | 4 (3) |
| 1^st^ PNC visit provider, n(%) |  |  |
| *HEW* | 186 (92) | 10 (7) |
| *Nurse* | 14 (7) | 112 (79) |
| *Health Officer/ Doctor* | 1 (0.5) | 17 (12) |
| Timing of first PNC visit, n(%) |  |  |
| *≤1 day post-delivery* | 28 (14) | 54 (38) |
| *2-7 days post-delivery* | 77 (38) | 32 (23) |
| *8-42 days post-delivery* | 98 (48) | 55 (39) |

Note: 14 women received their first PNC visit in an unknown location and are excluded from this table

**Supplementary Table 2** Association between household socioeconomic status and provider of first PNC visit and location of first PNC visit

|  | **SES Quintile n(%)** | | | | | **OR (95% CI)** | **p-value** |
| --- | --- | --- | --- | --- | --- | --- | --- |
|  | **1**  **Most Poor** | **2** | **3** | **4** | **5**  **Least Poor** |  |  |
| **Provider of first PNC visit** | | | | | | | |
| HEW | 40 (65) | 20 (50) | 40 (53) | 53 (66) | 50 (50) | 1.07 (0.88, 1.30) | 0.52 |
| Clinical Staff | 22 (35) | 20 (50) | 35 (47) | 27 (34) | 51 (50) |  |  |
| **Location of first PNC visit** | | | | | | | |
| Non-facility | 44 (71) | 18 (47) | 44 (61) | 49 (64) | 51 (53) | 1.10 (0.90, 1.35) | 0.33 |
| Facility | 18 (29) | 20 (53) | 28 (39) | 27 (36) | 45 (47) |  |  |
